# Effect of different interventions on the treatment of high-risk human papillomavirus infection: a systematic review and network meta-analysis

**DOI:** 10.3389/fmed.2024.1274568

**Published:** 2024-02-14

**Authors:** Dong-Yue Wang, Ying-Ying Cui, Wei-Wei Zhang, Meng-Si Fan, Ke-Xin Qiu, Li Yan

**Affiliations:** ^1^Department of Gynecology, The First Affiliated Hospital of Shandong First Medical University & Shandong Provincial Qianfoshan Hospital, Shandong First Medical University & Shandong Academy of Medical Sciences, Jinan, China; ^2^Key Laboratory of Laparoscopic Technology, department of Gynecology, The First Affiliated Hospital of Shandong First Medical University & Shandong Provincial Qianfoshan Hospital, Jinan, China; ^3^Department of Gynecology, Tengzhou Maternal and Child Health Hospital, Tengzhou, China; ^4^School of Public Health, Weifang Medical University, Weifang, China

**Keywords:** HR-HPV, conversion rate, comparison, network meta-analysis, randomized controlled trial

## Abstract

**Background:**

Persistent infection with high-risk human papillomavirus (HR-HPV) can lead to cervical intraepithelial neoplasia and cancer. At present, there is no medication that specifically targets HR-HPV infection.

**Objective:**

This study aimed to evaluate the effectiveness of different interventions in promoting HR-HPV regression using a MeSH meta-analysis method.

**Methods:**

A search for randomized controlled trials (RCTs) reporting different interventions for the treatment of HR-HPV infection included PubMed, Web of Science, Embase and Cochrane Library from the inception of the databases to March 8, 2023. Two researchers independently screened the articles, extracted data, and evaluated the quality. The literature that met the inclusion criteria was selected, the quality and risk of bias of the included studies were assessed according to the Cochrane 5.1 manual, and NMA was performed using Stata 16.0. The area under the cumulative ranking probability graph (SUCRA) represented the probability that each treatment would be the best intervention.

**Results:**

Nine studies involving 961 patients and 7 treatment options were included in the analysis. The results of the network meta-analysis indicated the following rank order in terms of promoting HR-HPV conversion: Anti-HPV biological dressing > vaginal gel > imiquimod > REBACIN® > interferon > probiotics > observation/placebo > Polyphenon E.

**Conclusion:**

Anti-HPV biological dressing treatment was found to be significantly effective in promoting HR-HPV conversion. However, further validation of the findings is necessary due to the limited number and quality of studies included in the analysis.

**Systematic review registration:**

https://www.crd.york.ac.uk/prospero/, identifier CRD42023413917.

## Introduction

Persistent high-risk human papillomavirus (HR-HPV) infection is a major risk factor for cervical intraepithelial neoplasia (CIN) and subsequent progression to cervical cancer (CC) (1, 2). Although HR-HPV infection is prevalent among women and typically resolves within a few months to 2 years (3), a minority of cases persist and pose an increased risk for progression to CIN 2 or higher (4). Accumulating evidence indicates that persistent HR-HPV infections are strongly associated with the development of HPV-driven malignancies (5). HR-HPV infection can progress to cervical cancer several years or decades later if the lesions are incompletely treated or even undetected or untreated. Kaiser Permanente Northern California Medical Care Plan (KPNC) data show that women with ASC-US, LSIL, or negative cytology with positive HPV16 or HPV18, or other high-risk HPV positivity for more than 1 year, may have an overall approximately 3.8% 5-year risk of a CIN3+ status (6). The effectiveness of treatment and clearance of patients with persistent HR-HPV infection has been the focus of controversy. The clearance of HR-HPV infection is commonly attributed to various factors, including viral subtype, host immunity, and environmental factors. Numerous researchers posit that administering pharmacological or physical therapy to HR-HPV-infected patients during follow-up can aid in HR-HPV clearance, alleviate the anxiety associated with follow-up alone, and facilitate lesion regression. Pharmacological treatment typically involves interferon and povidone-based suppositories. Studies have shown that pharmacological treatment can promote the clearance of HR-HPV by regulating the microenvironment and enhancing the immunity of the body (7, 8). Physical therapy includes freezing, laser, photodynamic and other new methods such as focused ultrasound and Non-invasive physical plasma (NIPP). Therefore, for patients with HR-HPV, supplemental medicine or physical therapy is necessary along with regular follow-up.

However, there is no specific medicine for the treatment of persistent HR-HPV infection worldwide, and the treatment options for HR-HPV vary among physicians; consequently, there is no conclusion as to which therapy is the most effective in the face of multiple treatment options. Although some articles have explored this topic using traditional meta-analysis, traditional meta-analysis can compare only two treatment options. In view of the limitations of previous meta-analyses, we conducted a network meta-analysis to determine the most effective approach for treating HR-HPV infection. NMA integrates direct and indirect evidence and enables the evaluation of multiple treatments in a single analysis (9). In this study, we determined the effectiveness of all interventions, as well as their ranking probabilities by summarizing the available evidence. The results of this investigation will provide evidence-based medical evidence for the treatment of HR-HPV infection.

## Methods

### Registration

Our study was conducted and reported according to the Preferred Reporting Items for Systematic Reviews and Meta-analyses (PRISMA-NMA) statement (10). This study is registered on the International Prospective Register of Systematic Review PROSPERO, number CRD42023413917. Since all analyses were founded on previously published research, neither ethical review nor patient permission are necessary.

### Search strategy and selection criteria

Our search strategy and selection criteria were conducted according to the guidelines of the new edition of the Cochrane Handbook for Systematic Reviews of Interventions (11). The search was conducted using PubMed, Web of Science, Embase and Cochrane Library from their inception dates to March 8, 2023. The research types were restricted to randomized controlled trials (RCTs), and the language was limited to English. We also retrieved articles from the reference lists and citations of the selected articles.

The search terms below were used individually or combined with each other: “HPV,” “Human Papillomavirus,” “Human Papilloma Virus,” “therapeutics,” “interferon,” “Baofukang suppository,” “lactobacillus,” “Anti-HPV biological protein dressing,” “nocardia rubra cell wall skeleton,” “photochemotherapy,” “cryotherapy,” “lasers, gas,” “ablation techniques,” “vaginal gel,” “polyphenon E,” “imiquimod,” “fluorouracil,” and “REBACIN®.”

### Inclusion and exclusion criteria

Articles that simultaneously satisfied all of the following requirements were ultimately included in our qualitative review (1): women who were diagnosed with cervical HR-HPV infection (12); women who were married or unmarried with a history of sexual activity, not limited by nationality, race or source of cases (2); trials that were RCTs only; and (3) English-only articles (4). The outcome indicator was the HPV clearance rate. Studies that had any of the following components were excluded (1): literature reviews, meta-analyses, systematic reviews, animal experiments, non-RCTs and literature with unavailable full text (2); repeatedly published studies or studies with duplicate data (3); inconsistent interventions, control measures, and outcome indicators.

### Literature selection

The selection of the literature was carried out independently by two researchers, and the results were cross-checked. The final selection of studies was agreed upon by all authors. If two researchers had inconsistent views on the selection of certain studies, the opinion of a third researcher was adopted to assist in the judgment and resolution.

### Quality assessment

The Cochrane Collaboration Network’s risk of bias assessment tool was utilized to evaluate the quality of the literature included in this study (13). The assessment entries consisted of several factors, including the method of random sequence generation, implementation of allocation concealment schemes, implementation of blinding, completeness of data, selective reporting bias and other biases. The evaluation process was conducted independently by two evaluators and cross-checked for accuracy.

### Data extraction

Two authors independently collected the relevant information. Endnote software was used for literature management, and Excel was used to generate tables for data extraction. The extracted literature data mainly included the title, name of the first author, publication date, sample size (experimental group/control group), diagnostic criteria, baseline comparability, intervention, outcome indicators and key elements of the risk of bias evaluation: randomized methods, allocation concealment, blinding, etc. In the event of incomplete data, the author of that study was contacted.

### Statistical analysis

Network meta-analysis (NMA) is an extension of traditional meta-analysis. NMA is an advanced form of meta-analysis that allows for the comparison of multiple interventions against each other rather than just comparing them to a single control group. The analysis involves using random or fixed effects models to determine the effect size for each intervention, with odds ratios (OR) used for dichotomous variables and standardized mean differences (SMDs) used for continuous variables. The results are typically presented with 95% confidence intervals (CIs). Stata 16.0 software was used to conduct the NMA analysis, with the data preprocessed using the network group command to create a network diagram of the evidence for each indicator. The area under the cumulative ranking (SUCRA) was obtained by ranking the efficacy of each indicator and plotting the probability ranking. The evidence network plot displays the number of patients for each intervention through the size of the dots, while the thickness of the lines between interventions represents the number of studies included. SUCRA expressed as a percentage indicates the effectiveness of the intervention, with larger percentages indicating higher efficacy and 0 indicating complete ineffectiveness (14, 15). If a closed loop existed between interventions, a nodal analysis model was utilized to compare the consistency of direct and indirect comparisons. Consistency was confirmed if *p* ≥ 0.05, and a statistical analysis was performed using a consistent model pair. Inconsistency was noted if *p* < 0.05, requiring a fitted inconsistency analysis. Funnel plots were created to identify any evidence of a small sample effect.

## Results

### Literature search and selection

**Figure 1:**
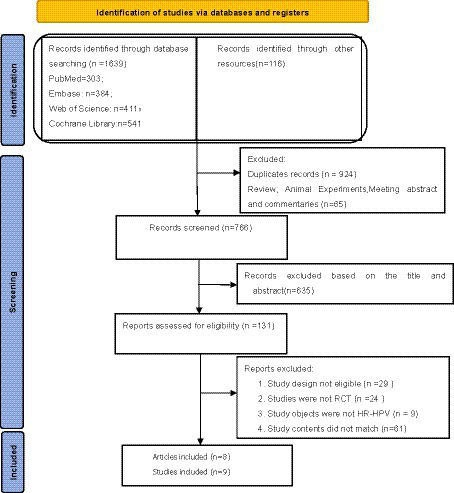
Literature review flowchart.

We obtained a total of 1755 articles according to the predetermined search strategy. We removed 924 duplicates that were identified by Endnote, and 65 studies, including literature reviews, meta-analyses, systematic reviews, meeting abstract and animal experiments, were excluded. Next, 635 articles were excluded by reading the titles and abstracts, and 123 articles were excluded according to the inclusion criteria and data integrity by reading the full text. Finally, a total of eight articles were included (16–23). A three-arm trial (19) and seven two-arm trials (including one with two parallel trials) (16–18, 20–23). A screening flow chart is presented in Figure 1.

In total, 961 patients were included, with 496 in the trial group and 465 in the control group. The analysis included seven interventions. The primary characteristics of the included studies are shown in Table 1.

**Table 1:** Characteristics of the included studies.

Study ID (First author)	Year	Interventions		Population		Age(mean ± SD)		First review time (Months)	Rate of HR-HPV clearance (%)	
		T	C	T	C	T	C		T	C
Major et al. (16)	2021	Vaginal Gel	Observation	65	76	33.3 ± 6.9	35.6 ± 8.8	3	49.2	10.53
Garcia et al. (17)	2014	PolyphenoE	Placebo	41	41	28.4	28.3	4	24.4	29.3
Grimm et al. (18)	2012	Imiquimod	Placebo	30	28	29.2 ± 6.1	31.8 ± 7.3	4	60.0	14.3
Zhou et al. (19)	2022	REBACIN® Interferon	Observation	35 36	35	18–70		4	68.6 25.0	20.0
Guo et al. (20)	2016	Anti-HPV biological dressing	Observation	38	37	43.6	45.0	3	60.5	13.5
Major et al. (21)	2021	Vaginal Gel	Observation	94	85	33.0 ± 6.7	35.5 ± 8.6	3	54.3	10.6
Yang et al. (22)	2019	REBACIN®	Observation	39	40	18–65		3	61.5	20.0
Yang et al. (22)	2019	REBACIN®	Observation	56	64	18–65		3	62.5	12.5
Ou et al. (23)	2019	Probiotics	Placebo	62	59	45.8 ± 10.8	43.8 ± 11.1	12	58.1	54.2

### Risk of bias assessment

**Figure 2:**
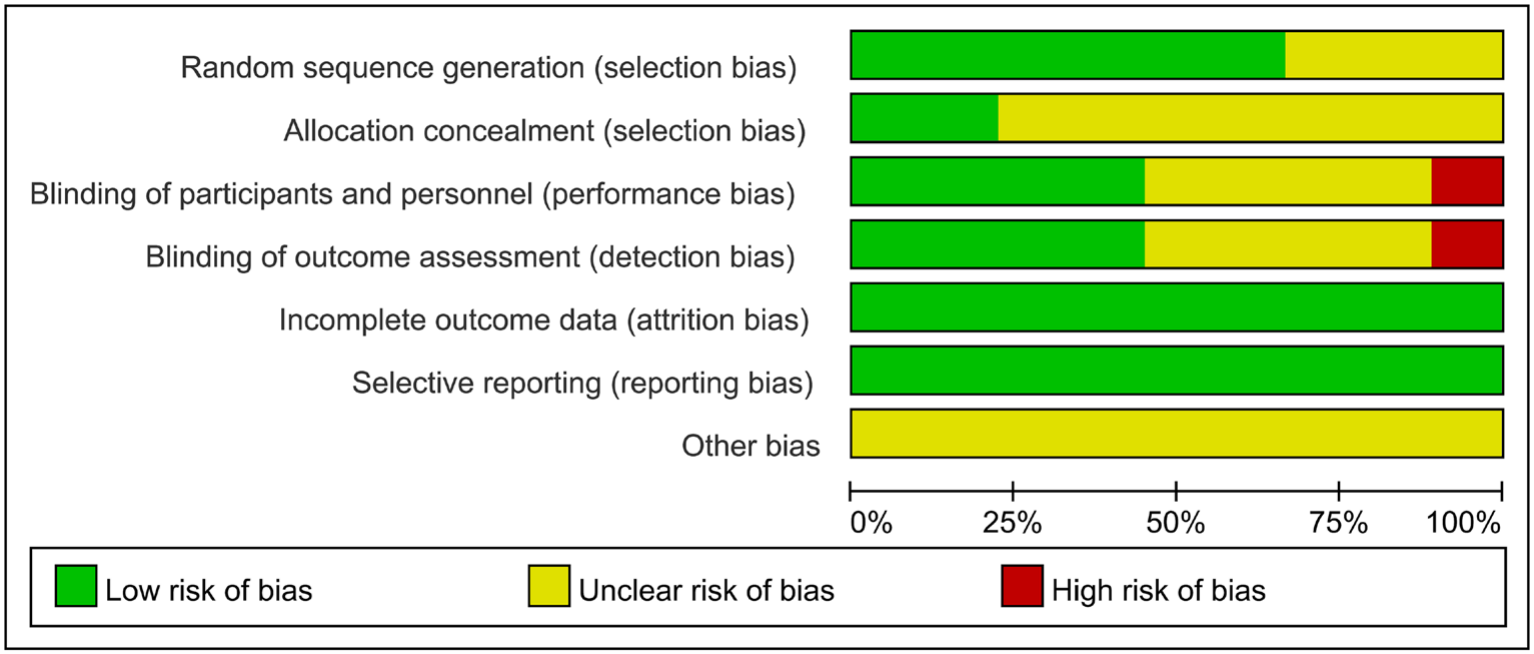
Detailed risk of bias analysis of the included trials.

A total of eight articles reporting on RCTs were included in the study, with one article reporting on two parallel studies (22), resulting in a total of nine studies analyzed. The general characteristics of the control and trial groups were comparable in all studies. Six studies reported using specific protocols for random sequence generation, with four using computerized random numbering (19, 22, 23), one using adaptive random allocation procedures (17), and one using randomized lists (16), all of which were rated as low risk. The remaining three studies did not report a specific method of randomization and only stated that the methods were random, so their risks were identified as unclear. Four studies used blinded methods and were rated as low risk (17, 18, 21, 23), whereas one studies did not use any blinding methods and were rated as high risk (16). Four studies did not state the blinding method and were rated as unclear risk (19, 20, 22). Nine studies did not have a registration protocol available; therefore, risk of bias was assessed through a comparison of the methodological description and results section in the RCT articles to evaluate data completeness and outcome reporting. One study (21) excluded one patient from the intervention group and six patients from the control group due to incomplete evaluation criteria. However, there was no significant difference in the number of excluded patients between groups (*p* = 0.280); thus, the study was rated as low risk. The remaining eight studies had no missing data and were also rated as low risk. Two studies (18, 23) reported allocation concealment, so their risks were rated as low risk; however, the allocation concealment in the remaining seven studies was unclear. No other biases were mentioned in any of the studies. The bias risk assessment of the included studies is shown in Figure 2.

### Evidence network

**Figure 3:**
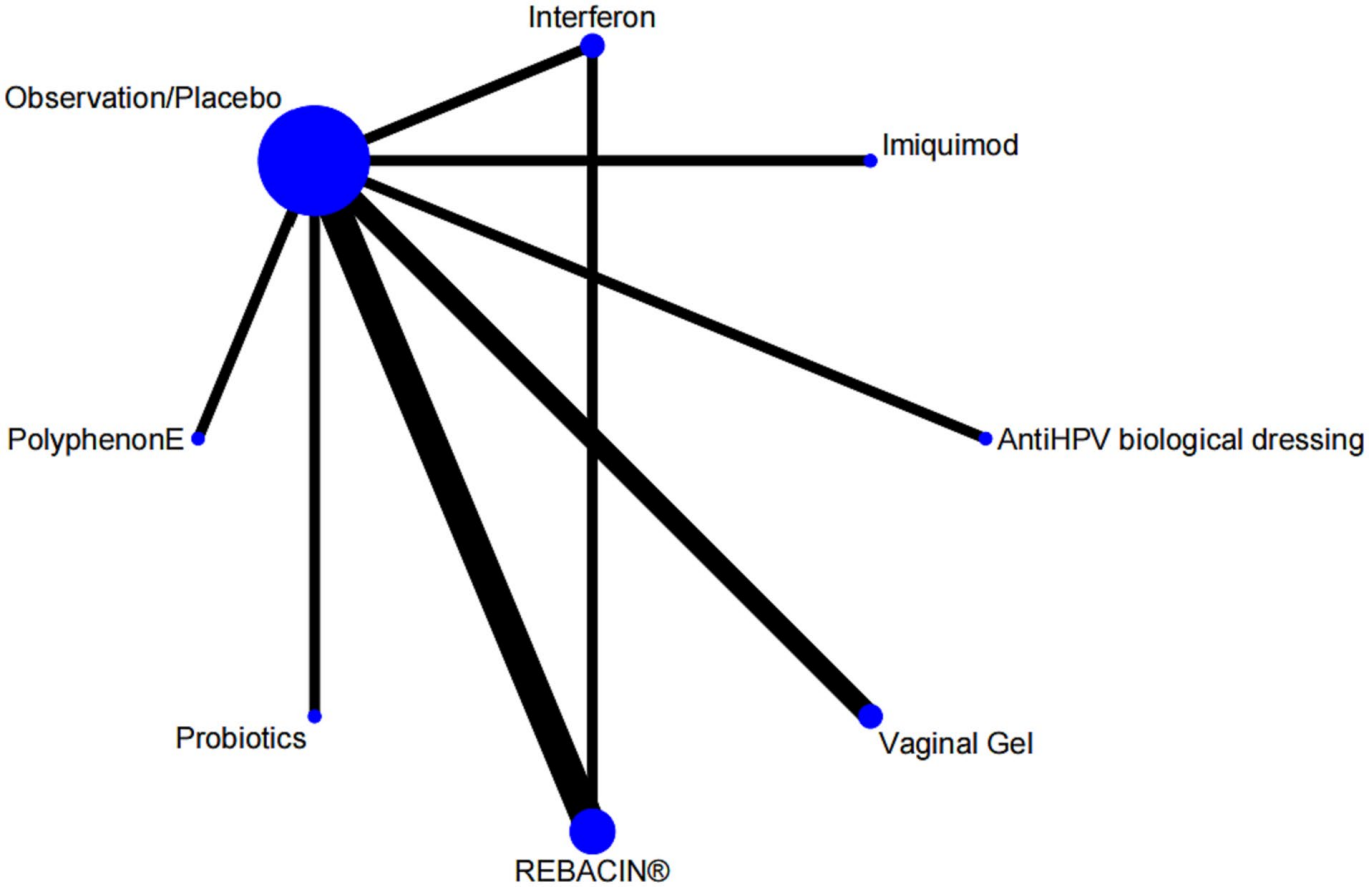
Network maps.

Nine studies reported overall effectiveness, with one being a three-arm study (24), and the rest being two-arm studies involving seven interventions. The evidence network was centered around the observation/placebo, as shown in Figure 3. There was one closed loops, including observation/placebo-interferon-REBACIN® therapy. The inconsistency test results showed that the *p* value and *Z* value for the closed loop of observation/placebo-interferon-REBACIN® therapy were 0.983 and 0.021, respectively, with a 95% CI of [0.00, 1.69]. The *p* values was greater than 0.05, indicating good consistency between direct and indirect comparisons in the closed loops.

### Efficacy comparison

**Figure 4:**
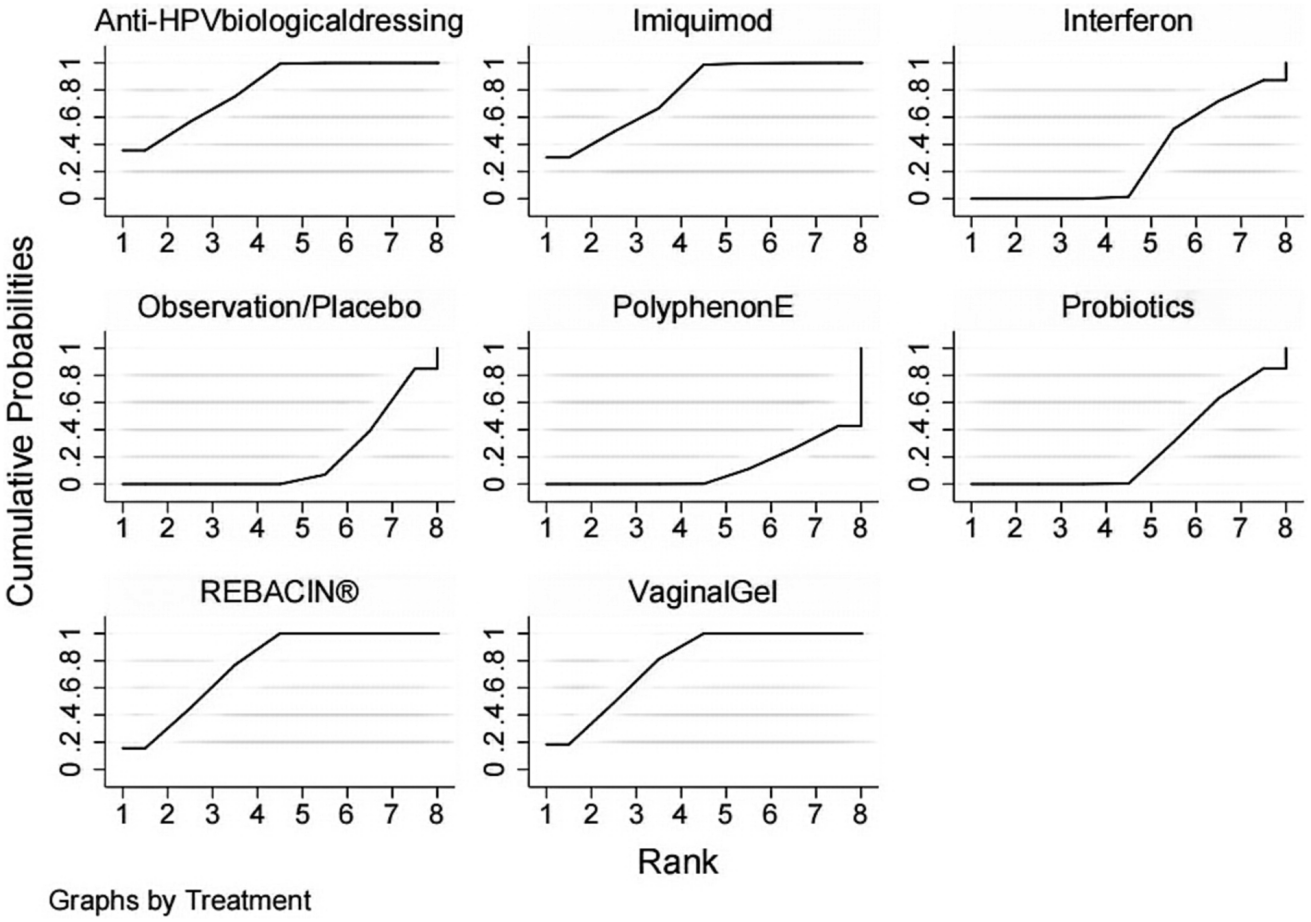
SUCRA probability ranking.

The included studies all reported the total clinical effectiveness rates, and the results of the network meta-analysis showed that, compared with interferon, interventions including Anti-HPV biological dressing [OR = 7.30, 95% CI (1.61,33.09)], vaginal gel [OR = 6.82, 95% CI (2.16, 21.54)], imiquimod [OR = 6.70, 95% CI (1.33, 33.85)], REBACIN® [OR = 6.58, 95% CI (2.52, 17.19)] were more effective in promoting the regression of HR-HPV infections. Compared with probiotics, interventions including Anti-HPV biological dressing [OR = 8.40, 95% CI (2.17, 32.47)], vaginal gel [OR = 7.85, 95% CI (3.09, 19.92)], imiquimod [OR = 7.70, 95% CI (1.77, 33.62)], and REBACIN® therapy[OR = 7.57, 95% CI (3.01, 19.02)] were more effective in promoting the regression of HR-HPV infections. Compared with observation/placebo, interventions including Anti-HPV biological dressing [OR = 9.81, 95% CI (3.12, 30.84)], vaginal gel [OR = 9.17, 95% CI (5.07, 16.58)], imiquimod [OR = 9.00, 95% CI (2.49, 32.57)], and REBACIN® therapy[OR = 8.84, 95% CI (4.97,15.74)] were more effective in promoting the regression of HR-HPV infections. Compared with PolyphenonE, interventions including Anti-HPV biological dressing [OR = 12.59, 95% CI (2.79, 56.83)], vaginal gel [OR = 11.76, 95% CI(3.74, 36.96)], imiquiod [OR = 11.54, 95% CI (2.29,58.16)], and REBACIN® therapy[OR = 11.34, 95% CI (3.64,35.37)] were more effective in promoting the regression of HR-HPV infections with statistical significance (*p* < 0.05). No significant differences were observed for the other intervention measures (*p* > 0.05), as shown in Table 2. The ranking of various intervention measure efficacy was achieved with the SUCRA line rank, which indicated that Anti-HPV biological dressing had the highest probability (SUCRA = 81.0%), followed by the vaginal gel(SUCRA = 78.4%), imiquimod (SUCRA = 77.8%), REBACIN® (SUCRA = 76.8%), interferon (SUCRA = 30.3%), probiotics (SUCRA = 25.7%), observation/placebo (SUCRA = 18.7%), and Polyphenon E (SUCRA = 11.4%). The results of the SUCRA probability ranking are shown in Figure 4.

**Table 2:** Network meta-analysis of the effectiveness [OR (95% CI)].

Anti-HPV biological dressing							
1.07 (0.29,3.89)	Vaginal gel						
1.09 (0.19,6.10)	1.02 (0.25,4.20)	Imiquimod					
1.11 (0.31,4.00)	1.04 (0.45,2.37)	1.02 (0.25,4.17)	REBACIN®				
7.30 (1.61,33.09)	6.82 (2.16,21.54)	6.70 (1.33,33.85)	6.58 (2.52,17.19)	Interferon			
8.40 (2.17,32.47)	7.85 (3.09,19.92)	7.70 (1.77,33.62)	7.57 (3.01,19.02)	1.15 (0.34,3.89)	Probiotics		
9.81 (3.12,30.84)	9.17 (5.07,16.58)	9.00 (2.49,32.57)	8.84 (4.97,15.74)	1.34 (0.50,3.60)	1.17 (0.57,2.40)	Observation/placebo	
12.59 (2.79,56.83)	11.76 (3.74,36.96)	11.54 (2.29,58.16)	11.34 (3.64,35.37)	1.72 (0.43,6.92)	1.50 (0.44,5.05)	1.28 (0.48,3.42)	PolyphenonE

### Publication bias

**Figure 5:**
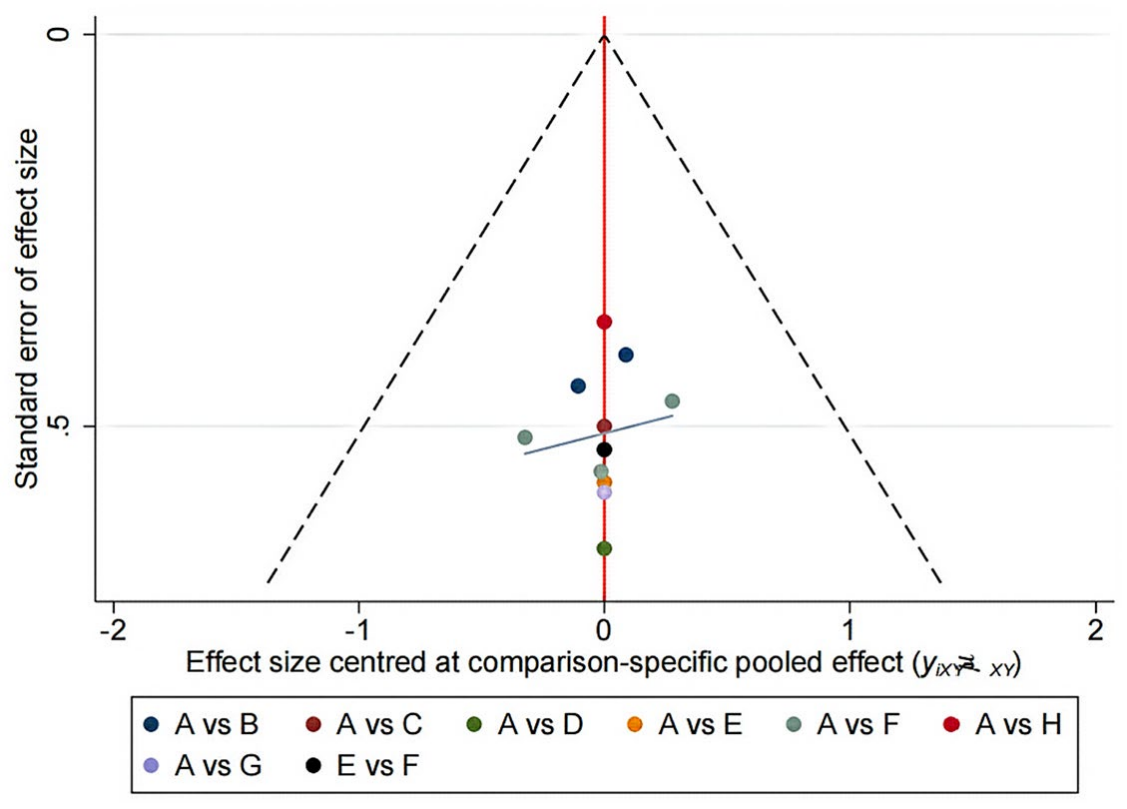
Funnel plot.

The small sample size effects of each outcome indicator were tested using Stata 16.0, and a “comparison-adjustment” funnel plot was created, as shown in Figure 5. The results showed that all studies were roughly distributed on both sides of the center line, indicating a small possibility of publication bias.

## Discussion

In recent years, the incidence and mortality of cervical cancer in developed countries have been gradually reduced by the increasing popularity of cervical cancer vaccines and cervical cancer screening, but the incidence and mortality of cervical cancer in China are increasing, while the age of onset is decreasing, especially in rural areas where vaccines and screening are not yet widespread. About 59,000 women died of cervical cancer in China in 2020, accounting for 17.3% of the global total (24). It is clear that HR-HPV infection is an independent risk factor for cervical cancer, and Thomsen LT et al. (25) stated that the absolute risk of progression to CIN3 or cancer within 12 years was estimated to be 47.4% when both follow-ups were positive for HR-HPV at an interval of 2 years. There are no specific medicine for HPV infection, and how to promote a more rapid HPV conversion has been a challenge for physicians and patients. Therefore, this study provides evidence-based medical evidence for the treatment of HR-HPV infection by comparing seven treatment regimens commonly used in clinical practice to promote regression of HR-HPV infection by means of a reticulated meta-analysis, with a view to providing reference implications for clinical research and practice.

The current clinical evaluation of the efficacy of various interventions for HR-HPV infection is mainly based on the HR-HPV conversion rate. This study finally included nine RCTs in a reticulated meta-analysis to compare the efficacy of seven interventions and observation/placebo. The results showed that Anti-HPV biological dressing had the best efficacy in promoting HR-HPV conversion, with the order of efficacy being Anti-HPV biological dressing > vaginal gel > imiquimod > REBACIN® > interferon > probiotics > observation/placebo > Polyphenon E.

In our study, The Anti-HPV biological dressing was the most effective therapy. Developed independently in China, the Anti-HPV biological dressing is a new type of virus inhibitor whose main components include JB protein, a carbomer and tea polyphenol. JB protein changes the protein conformation of HPV by the mechanism of positive and negative charges to accelerate the inactivation of HPV; the carbomer has strong adhesion, which can isolate the infected wound from normal tissue and promote the repair and regeneration of the wound. Tea polyphenols can improve local immunity. Guo et al. (20) showed that 60.5% of HR-HPV-positive patients turned negative after 3 months of treatment with an Anti-HPV biological dressing. Similarly, a study showed that the Anti-HPV biological dressing reduced the viral load by 88.4% (26).

The vaginal gel mentioned in this article was SAM vaginal gel which is the second effective medicine. SAM vaginal gel has specific powerful antioxidant and adsorbent properties that bind potential pathogens (16, 21). Major et al. (21) showed that 3 months after SAM vaginal gel treatment, 54% of the participants were HR-HPV negative, and 75% had improved cytologic results (determined as complete resolution of lesions or change to lower-grade lesions). Imiquimod is an immunomodulator whose antiviral and antitumor abilities are not direct, but work by inducing the body’s keratin-forming cells to produce interferon, tumor necrosis factor, and interleukins. Cokan et al. (27) reported a regression rate of up to 51.9% for imiquimod in the treatment of cervical HSIL (CIN 2–3), however, in the CIN2 p16-positive subgroup, the therapeutic efficacy of imiquimod was as high as 73.9% (28).

Interferon and probiotics are the two most commonly used medicine for the treatment of HPV infection in China, but in this study, they ranked low in terms of therapeutic efficacy. Inclusion studies have shown that the conversion rate of HR-HPV infection using topical administration of interferon can reach 25–35% (19, 29, 30). Veronique et al. (31) found that probiotic treatment for 3 months resulted in an HR-HPV conversion rate of 25%. Qu et al. (23) reported a 58.1% conversion rate for 12 months of probiotic treatment. The efficacy was not significant compared to other treatment regimens. Considering that this difference may be due to the number of included studies and sample size, etc., more prospective, high-quality studies are needed in the future to further confirm their efficacy.

In addition to medication, physical therapy is the primary treatment for HR-HPV infection. Traditional physical therapy includes cryotherapy, ablation and photodynamic therapy. Cryotherapy works by causing local circulation disorders through the low temperature of liquid nitrogen, which leads to tissue necrosis. Ablation therapy uses electromagnetic waves to target the diseased tissue, causing coagulation, denaturation and further necrosis, and the thermal effect of coagulation activates the body’s immune system to clear the HPV. Photodynamic therapy uses a laser at a certain frequency to produce a photochemical reaction with cells that have absorbed the photosensitizer, generating a large amount of reactive oxygen species and leading to apoptosis and necrosis. For histologically confirmed CIN2/3 patients, the WHO currently suggests excisional treatments and under certain conditions (e.g., fully visible transformation zone and CIN lesions) destructive treatments like laser-, thermal-, and cryo-ablationcan also be considered (32). Pinder et al. (33) reported 40, 42 and 47% conversion rates of HR-HPV 6 months after cryotherapy, thermal ablation and LEEP, respectively.

In recent years, new non-invasive physical therapy techniques have been gradually developed. Examples include focused ultrasound, non-invasive physical plasma (NIPP). Focused ultrasound is a new, noninvasive physical therapy technology. By converting the mechanical effect into a thermal effect and a cavitation effect, the lesion undergoes coagulative necrosis, thus achieving the purpose of noninvasive treatment. Fu et al. (34) reported that the HPV conversion rate was 85.7% at 3 months after focused ultrasound treatment. Qin et al. (35) found that the clearance rate of HPV was 94.1% after 12 months of focused ultrasound treatment. The NIPP procedure was carried out using a modular high frequency device, it affects cellular processes such as cell growth, metabolism, cell cycle, and DNA integrity mainly through reactive oxygen species (ROS) production (36, 37). Which is highly available in surgical facilities, however, fewer studies have been done in cervical precancerous lesions and cervical cancer. Martin Weiss et al. showed a two-fold reduction in overall high-risk HPV infections 6 months after NIPP treatment (32). However, in our study, no physical therapy program was identified that met the inclusion criteria.

### Innovation and limitations of this study

In our study, the efficiency of comparing multiple interventions to promote HR-HPV conversion was assessed with the help of reticulated meta-analysis, which is an extension of traditional meta-analysis to achieve an indirect comparison of multiple similar studies and thus greatly improve the value of individual RCTs. However, there are certain limitations (1): some literature is of low quality, does not describe random methods and without using blind methods (2); the number of studies and sample size included in each intervention protocol varied widely, so bias may have existed between different intervention protocols; and (3) the frequency, duration of treatment, and follow-up time of the intervention protocols included in this study were not uniform, which may have affected the results. Therefore, higher-quality RCTs are needed to further assess the efficacy of each treatment.

## Conclusion

In summary, among the 7 interventions, Anti-HPV biological dressing was the most effective in promoting the conversion of HR-HPV infection, with interferon and probiotics, which are widely used clinically, ranking low. Even the best medicine, Anti-HPV biological dressing, have a conversion rate of only 60.5%. In recent years, more and more physical therapy has been applied to patients, however, large, high-quality randomized controlled studies should be done in the future to verify their true efficacy.

### Recommendations

In summary, the author provides recommendations for conducting future RCTs. First, the research design should conform to the internationally recognized SPIRIT declaration (38), strictly follow the PICO principle, and register the research plan before the trial, and the final report should be standardized according to the CONSORT declaration (39). Second, due to the long duration, low regression rate, and long treatment cycle of HR-HPV infection, the outcome indicators should also consider patient compliance and the incidence of adverse reactions caused by the treatment plan. Third, cost–benefit analysis should be added to the efficacy evaluation, facilitating a reasonable selection of treatment options based on efficacy and economic considerations in clinical practice.

## Author contributions

D-YW: Data curation, Formal analysis, Investigation, Writing – original draft. Y-YC: Conceptualization, Methodology, Project administration, Writing – review & editing. W-WZ: Data curation, Software. M-SF: Data curation, Software. K-XQ: Data curation, Software. LY: Resources, Writing – review & editing. All authors contributed to the article and approved the submitted version.
