# Supplementary material for: Effect of different interventions on the treatment of high-risk human papillomavirus infection: a systematic review and network meta-analysis
Source: Front Med (Lausanne). 2024 Feb 14;11:1274568. doi: 10.3389/fmed.2024.1274568 (PMC10899477; doi:10.3389/fmed.2024.1274568)
Supplement: Supplementary file 1 [file Data_Sheet_1.PDF]

Pubmed Search strategy; searched on March 8, 2023.

#1 Search "Human Papillomavirus Viruses"[Mesh]

#2 Search(Human Papillomavirus Virus[Title/Abstract]) OR (Papillomavirus Virus, Human[Title/Abstract]) OR (Virus, Human Papillomavirus[Title/Abstract]) OR (Human Papillomavirus[Title/Abstract]) OR (Human Papillomaviruses[Title/Abstract]) OR (HPV, Human Papillomavirus Viruses [Title/Abstract]) OR (Human Papilloma Virus[Title/Abstract]) OR (Human Papilloma Viruses[Title/Abstract]) OR (Papilloma Virus, Human[Title/Abstract]) OR (Virus, Human Papilloma[Title/Abstract]) OR (HPV Human Papillomavirus[Title/Abstract]) OR (HPV Human Papillomaviruses [Title/Abstract]) OR (Human Papillomavirus, HPV[Title/Abstract]) OR (Human Papillomaviruses, HPV[Title/Abstract])

#3 Search ("High Risk"[Mesh])

#4 Search ("Randomized Controlled Trial"[Mesh])

#5 Search(randomized[Title/Abstract])OR (placebo[Title/Abstract])

#6 Search "Therapeutics"[Mesh]

#7 Search(Therapeutic[Title/Abstract]) OR (Therapy[Title/Abstract]) OR (Therapies[Title/Abstract]) OR (Treatment[Title/Abstract]) OR (Treatments[Title/Abstract])

#8 Search "Interferons"[Mesh]

#9 Search (Interferon[Title/Abstract])

#10 Search "Baofukang suppository "[Mesh]

#11 Search "Lactobacillus"[Mesh]

#12 Search "Probiotics"[Mesh]

#13 Search (Probiotic[Title/Abstract])

#14 Search "Anti-HPV Biological Protein Dressing"[Mesh]

#15 Search "Nocardia Rubra Cell Wall Skeleton"[Mesh]

#16 Search "Photochemotherapy"[Mesh]

#17 Search(Photochemotherapies[Title/Abstract]) OR (Photodynamic Therapy[Title/Abstract]) OR (Therapy, Photodynamic[Title/Abstract]) OR (Photodynamic Therapies[Title/Abstract]) OR (Therapies, Photodynamic[Title/Abstract])

#18 Search "Cryotherapy"[Mesh]

#19 Search(Cryotherapies [Title/Abstract]) OR (Cold Therapy[Title/Abstract]) OR (Cold Therapies[Title/Abstract]) OR (Therapies, Cold[Title/Abstract]) OR (Therapy, Cold[Title/Abstract])

#20 Search “Lasers, Gas”[Mesh]

#21 Search(Gas Laser[Title/Abstract]) OR (Laser, Gas[Title/Abstract]) OR (Gas Lasers[Title/Abstract]) OR (Carbon Dioxide Lasers[Title/Abstract]) OR (Carbon Dioxide Laser[Title/Abstract]) OR (Dioxide Laser, Carbon[Title/Abstract]) OR (Dioxide Lasers, Carbon[Title/Abstract]) OR (Laser, Carbon Dioxide[Title/Abstract]) OR (Lasers, CO2[Title/Abstract]) OR (CO2 Lasers[Title/Abstract]) OR (CO2 Laser[Title/Abstract]) OR (Laser, CO2[Title/Abstract]) OR (Lasers, Carbon Dioxide[Title/Abstract])

#22 Search “microwave therapy ”[Mesh]

#23 Search “Ablation Techniques ”[Mesh]

#24 Search(Ablation Technique[Title/Abstract]) OR (Technique, Ablation[Title/Abstract]) OR (Techniques, Ablation[Title/Abstract])

#25 Search “Vaginal Creams, Foams, and Jellies ”[Mesh]

#26 Search((Vaginal Gel[Title/Abstract]) OR (Gel, Vaginal[Title/Abstract]) OR (Gels, Vaginal[Title/Abstract]) OR (Vaginal Gels[Title/Abstract])

#27 Search “Polyphenon E ”[Mesh]

#28 Search “Imiquimod ”[Mesh]

#29 Search “Fluorouracil ”[Mesh]

#30 Search(5FU[Title/Abstract]) OR (5-FU[Title/Abstract]) OR (5-Fluorouracil[Title/Abstract]) OR (5 Fluorouracil[Title/Abstract]) OR (Fluoruracil[Title/Abstract]) OR (5-Fluorouracil-Biosyn[Title/Abstract]) OR (5 Fluorouracil Biosyn[Title/Abstract]) OR (Fluoro-Uracile[Title/Abstract]) OR (Fluoro Uracile[Title/Abstract])

#31 Search “rebacin ”[Mesh]

#32 Search(Biological Products[Title/Abstract])

#33 #1 OR #2

#34 #3 and #33

#35 #4 OR #5

#36 #34 AND #35

#37 #6 OR #7 OR #8 OR #9 OR #10 OR #11 OR #12 OR #13 OR #14 OR #15 OR #16 OR #17 OR #18 OR #19 OR #20 OR #21 OR

#22 OR #23 OR #24 OR #25 OR #26 OR #27 OR #28 OR #29 OR #30 OR #31 OR #32

#38 #36 AND #37
