# Supplementary material for: Effect of different interventions on the treatment of high-risk human papillomavirus infection: a systematic review and network meta-analysis
Source: Front Med (Lausanne). 2024 Feb 14;11:1274568. doi: 10.3389/fmed.2024.1274568 (PMC10899477; doi:10.3389/fmed.2024.1274568)
Supplement: Supplementary file 2 [file Data_Sheet_2.PDF]

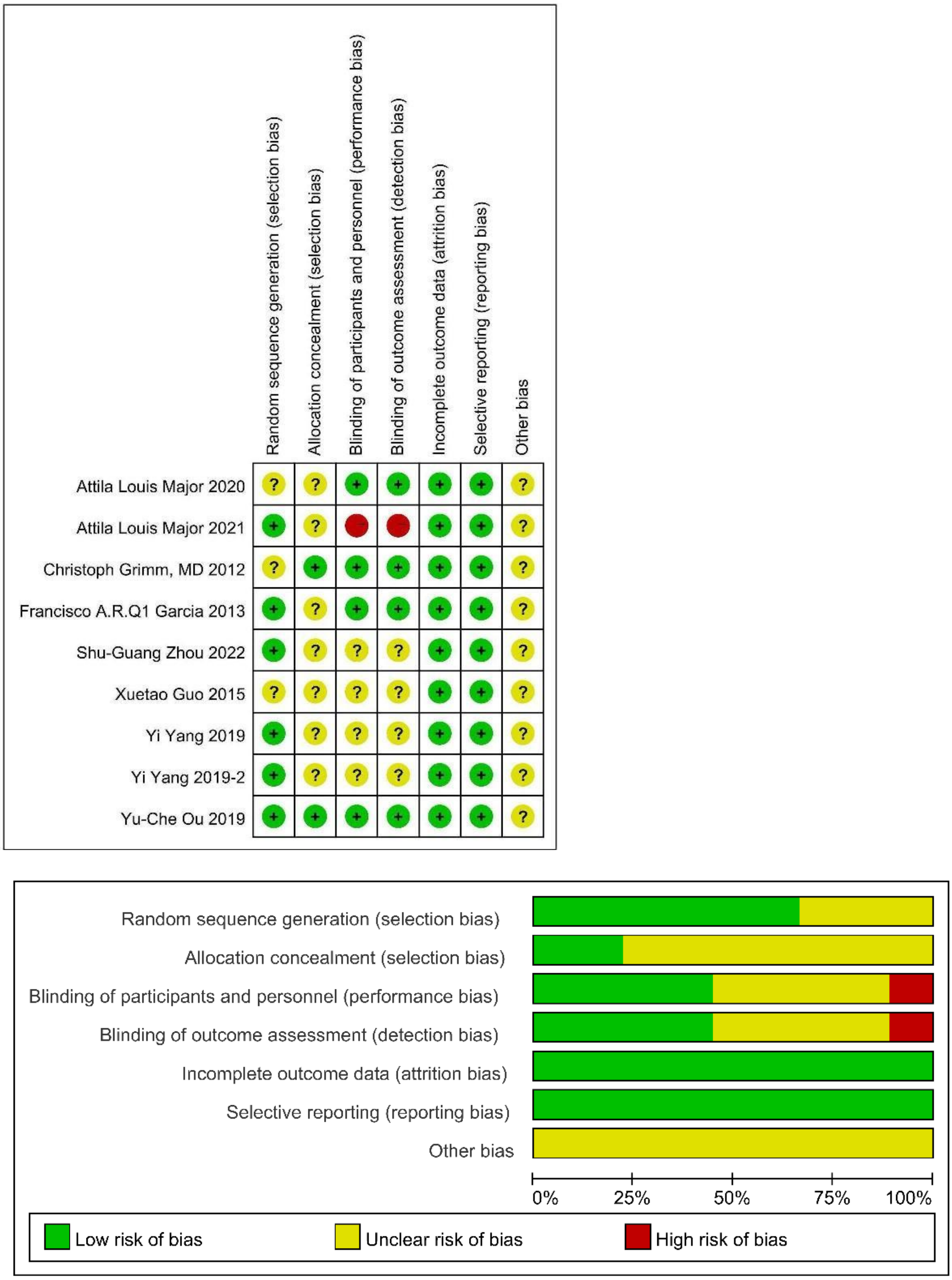

Quality assessment for the bias risk of trials.
